# Efficacy and Safety of Tirzepatide in Japanese Participants With Obesity: A Subpopulation Analysis of the SURMOUNT‐1 Trial

**DOI:** 10.1002/oby.70131

**Published:** 2026-01-30

**Authors:** Yasushi Ishigaki, Masamichi Yamada, Tomotaka Shingaki, Tomonori Oura, Iichiro Shimomura

**Affiliations:** ^1^ Division of Diabetes, Metabolism and Endocrinology, Department of Internal Medicine Iwate Medical University Iwate Japan; ^2^ Tokyo Center Clinic Tokyo Japan; ^3^ Japan Drug Development and Medical Affairs Eli Lilly Japan K.K. Kobe Hyogo Japan; ^4^ Department of Metabolic Medicine Osaka University Graduate School of Medicine Osaka Japan

**Keywords:** Japan, obesity, overweight, tirzepatide

## Abstract

**Objective:**

This prespecified subpopulation analysis aimed to assess the efficacy and safety of once‐weekly tirzepatide versus placebo alongside lifestyle intervention in Japanese adults with obesity or overweight.

**Methods:**

Data from 102 Japanese adults in the SURMOUNT‐1 trial with BMI ≥ 30 kg/m^2^ or ≥ 27 kg/m^2^ and ≥ 1 weight‐related comorbidity were analyzed. Coprimary endpoints were mean percent change in body weight and the proportion of participants who achieved ≥ 5% body weight reduction at week 72.

**Results:**

Participants in the tirzepatide 5‐, 10‐, and 15‐mg groups had a statistically significantly greater (all *p* < 0.001) least squares mean (standard error) percent change in body weight compared with those in the placebo group: −12.0% (1.7%), −22.4% (1.7%), −22.1% (1.6%), and −0.3% (1.6%), respectively. Overall, 91.7%, 100%, and 96.6% of participants in the tirzepatide 5‐, 10‐, and 15‐mg groups, respectively, had ≥ 5% weight reduction at week 72, compared with 15.4% in the placebo group. Significant improvements in cardiometabolic measures were also observed with tirzepatide at week 72 compared to placebo. No new safety concerns were identified.

**Conclusions:**

Once‐weekly treatment with tirzepatide demonstrated significant reductions in body weight and prespecified cardiometabolic measures compared with placebo in Japanese adults with obesity or overweight.

**Trial Registration:**

ClinicalTrials.gov identifier: NCT04184622 https://clinicaltrials.gov/study/NCT04184622

## Study Importance

- What is already known?
  ○ The global, multicenter, phase 3 SURMOUNT‐1 trial demonstrated substantial and sustained body weight reductions in adults with obesity or overweight treated with once‐weekly tirzepatide (5, 10, or 15 mg) or placebo over 72 weeks.
  ○ Improvements in all prespecified cardiometabolic measures, including lipids, cholesterol, and systolic/diastolic blood pressure, were also observed with tirzepatide treatment.
- What does this study add?
  ○ In this subpopulation analysis of Japanese adults with obesity or overweight from SURMOUNT‐1, treatment with tirzepatide (5, 10, or 15 mg) for 72 weeks resulted in significant reductions in body weight and prespecified cardiometabolic measures compared with placebo
  ○ All doses of tirzepatide were generally well tolerated, with no new safety concerns identified.
- How might these results change the direction of research or the focus of clinical practice?
  ○ These findings add to the limited data on tirzepatide in Asian populations with obesity or overweight, including Japanese individuals, and are of clinical importance as Japanese individuals have a higher risk of obesity‐related health disorders at a lower BMI than Caucasians.
  ○ Considering the chronic nature of obesity and its related health issues, these findings support the longer‐term studies of tirzepatide in Japanese individuals with obesity or overweight.

## 1 Introduction

Overweight and obesity are associated with increased risks of morbidity, including type 2 diabetes (T2D), cardiovascular disease, dyslipidemia, and sleep disorders [1]. The Japanese Society for the Study of Obesity criteria define obesity in Japanese individuals as having a body mass index (BMI) ≥ 25 kg/m^2^ [2]. Asian populations, including Japanese individuals, have a higher risk of obesity‐related health disorders at lower BMIs compared with Caucasians [2] due to a greater visceral fat accumulation [3]. Japanese individuals also tend to accumulate more visceral fat than those of European ancestry with a similar BMI [2, 4, 5]. Given the high susceptibility of Japanese individuals to weight‐related health conditions, obesity is a key focus of Japanese public health policy [6].

Obesity is a chronic condition influenced by a range of factors, including genetic, metabolic, environmental, and behavioral components [7]. For people with obesity, achieving long‐term weight loss is challenging, and most individuals tend to regain the weight they lose over time [8]. In conjunction with lifestyle interventions, several clinical guidelines recommend pharmacotherapy for people with obesity or overweight with weight‐related comorbidities [7].

Tirzepatide is a long‐acting dual glucose‐dependent insulinotropic polypeptide and glucagon‐like peptide‐1 receptor agonist [9] approved in the United States and Europe for the treatment of T2D in adults and for chronic weight management in adults with obesity or overweight and at least one weight‐related condition. Additionally, tirzepatide was approved for the treatment of obesity disease in Japan based on the findings from the phase 3 SURMOUNT clinical trial program. The global SURMOUNT clinical trial program assessed the efficacy and safety of tirzepatide to reduce excess body weight and maintain long‐term weight reduction as a treatment for people with overweight or obesity. As part of the SURMOUNT program, the SURMOUNT‐J trial assessed the efficacy and safety of tirzepatide (10 or 15 mg) compared with placebo in 225 Japanese adults with obesity disease (defined as BMI ≥ 27 kg/m^2^ accompanied by two or more obesity‐related health problems or ≥ 35 kg/m^2^ accompanied by at least one obesity‐related health problem excluding diabetes) [10]. Findings from the SURMOUNT‐J trial demonstrated statistically significant and clinically meaningful reductions in body weight following 72 weeks of tirzepatide treatment compared with placebo, with a safety profile consistent with observations in global populations [10].

The phase 3 SURMOUNT‐1 trial examined the efficacy and safety of once‐weekly tirzepatide 5, 10, and 15 mg compared with placebo in participants with obesity or overweight and without T2D from nine countries including Japan [11]. In all tirzepatide treatment groups, substantial and sustained body weight reductions were observed over 72 weeks. Furthermore, improvements in all prespecified cardiometabolic measures were observed with tirzepatide treatment. The safety profile of tirzepatide in SURMOUNT‐1 was generally similar to other incretin‐based therapies for weight management, with the most frequent adverse events (AEs) being mild to moderate gastrointestinal events that primarily occurred during the dose‐escalation period [11]. Data on tirzepatide in Japanese patients with obesity are limited, and the efficacy and safety of the 5‐mg dose have not been investigated in this population. This analysis adds to the limited data by evaluating the efficacy and safety of once‐weekly tirzepatide in Japanese participants with obesity using data from the SURMOUNT‐1 trial.

## 2 Methods

### 2.1 Participants

This was a subpopulation analysis of Japanese participants from the SURMOUNT‐1 trial (NCT04184622), conducted in nine countries, that investigated the efficacy and safety of once‐weekly doses of tirzepatide 5, 10, and 15 mg compared with placebo on weight reduction over a 72‐week period. The SURMOUNT‐1 trial randomized 2539 participants, of whom 70.6% (*n* = 1792) were White, 10.9% (*n* = 276) were Asian, 9.1% (*n* = 231) were American Indian or Alaskan Native, and 7.9% (*n* = 201) were Black or African American. The Japanese population included in this analysis constituted 4% (*n* = 102) of the overall population.

Eligible participants were aged ≥ 18 years with one or more self‐reported unsuccessful dietary efforts to lose weight and had BMI ≥ 30 kg/m^2^ or ≥ 27 kg/m^2^ and at least one weight‐related comorbidity (obstructive sleep apnea, hypertension, dyslipidemia, or cardiovascular disease). Individuals were excluded if they had diabetes, received treatment with medications or remedies that may have resulted in weight gain or loss within 3 months before randomization, reported a change in body weight of > 5 kg within 3 months before screening, had obesity induced by other endocrinologic disorders, or were diagnosed with monogenetic or syndromic forms of obesity or had a history of chronic or acute pancreatitis, family or personal history of medullary thyroid carcinoma or multiple endocrine neoplasia syndrome type 2, history of significant active or unstable major depressive disorder or other severe psychiatric disorders within the last 2 years, or any lifetime history of a suicide attempt.

### 2.2 Trial Design and Procedures

A full description of the study design and outcomes of the SURMOUNT‐1 trial has been published [11]. Briefly, SURMOUNT‐1 was a phase 3, multicenter, randomized, placebo‐controlled, double‐blind study of the safety and efficacy of once‐weekly tirzepatide compared with placebo for weight management when used in conjunction with a reduced‐calorie diet and increased physical activity. After a 2‐week screening period, participants were randomized 1:1:1:1 to receive once‐weekly tirzepatide 5 mg, tirzepatide 10 mg, tirzepatide 15 mg, or placebo. A computer‐generated random sequence using an interactive web‐response system randomly assigned participants to treatment groups, with randomization stratified by participants' prediabetes status, country, and sex. For participants randomly assigned to receive tirzepatide, the starting dose was 2.5 mg once weekly via subcutaneous injection. The dosage was increased in 2.5‐mg increments every 4 weeks until the assigned study dose was achieved (5, 10, or 15 mg). The dose‐escalation period continued for up to 20 weeks. Participants in the placebo group received a matching once‐weekly placebo. After randomization, participants completed the 72‐week treatment period. Participants with prediabetes at randomization completed an additional 2‐year treatment period, whereas participants without prediabetes proceeded to a 4‐week safety follow‐up period. Participants were advised to undergo a lifestyle intervention comprising a hypocaloric diet with a 500‐cal deficit that was individually calculated and an increase in physical activity by 150 min per week. Lifestyle counseling was administered throughout the entire study.

The study protocol was approved by local institutional review boards, and the trial complied with the International Council on Harmonisation of Technical Requirements for Registration of Pharmaceuticals for Human Use, Good Clinical Practice guidelines, and the Declaration of Helsinki. All participants provided written informed consent prior to trial participation.

### 2.3 Outcomes

The coprimary endpoints of the global SURMOUNT‐1 trial were the mean percent change in body weight from baseline to week 72 and the percentage of participants achieving at least a 5% body weight reduction at week 72. Key secondary and prespecified cardiometabolic endpoints included mean change in body weight from baseline to week 20, percentage of participants achieving ≥ 10%, ≥ 15%, and ≥ 20% body weight reduction at week 72, and change from baseline to week 72 in waist circumference, triglycerides, high‐density lipoprotein cholesterol (HDL‐C), non‐HDL‐C, systolic blood pressure (BP), and glycated hemoglobin. Additional endpoints included change from baseline to week 72 in diastolic BP, low‐density lipoprotein cholesterol, very low‐density lipoprotein cholesterol, total cholesterol, and free fatty acids. Safety endpoints included treatment‐emergent AEs, serious AEs, and deaths.

### 2.4 Statistical Analysis

Comprehensive details on the statistical analysis methods have been published [11]. Efficacy and safety were assessed using the modified intention‐to‐treat population, which comprised all randomized participants who received at least one dose of the study drug. The efficacy analyses were guided by the efficacy estimand, defined as the average treatment effect of tirzepatide relative to placebo at 72 weeks. This was considered as an adjunct to a reduced‐calorie diet and increased physical activity, assuming that the randomized participants had received their assigned treatment for the entire 72‐week treatment duration. For these analyses, the efficacy analysis set was used.

Summary statistics for categorical measures (including categorized continuous measures) included sample size, frequency, and percentage. A mixed model for repeated measures was used to compare treatment groups relative to continuous measurements. This model included terms for treatment group, visit, sex, prediabetes status at randomization, and treatment‐by‐visit interaction as fixed effects, with the baseline measurement as a covariate. A logistic regression model was conducted to analyze the percentage of participants who achieved at least a 5% reduction in body weight from randomization at the 72‐week visit. The model included terms for treatment group, sex, and prediabetes status at randomization as fixed effects, with baseline body weight as a covariate. Postbaseline missing data were imputed via mixed model for repeated measures predictions and dichotomized. Tests of treatment effects were conducted at a two‐sided alpha level of 0.05, and two‐sided 95% confidence intervals (CI) were calculated. No type I error control was applied.

## 3 Results

### 3.1 Study Participants

There were 102 Japanese participants enrolled in the SURMOUNT‐1 trial. Data in the current analysis exclude 27 screened and 22 randomized participants because of the exclusion of one study site due to a regulatory noncompliance issue. Of the 102 participants, 24 received tirzepatide 5 mg, 22 received tirzepatide 10 mg, 29 received tirzepatide 15 mg, and 27 received placebo.

**FIGURE 1:**
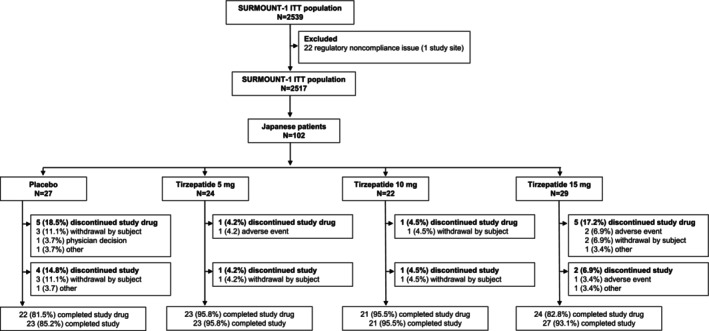
Participant disposition. Flow diagram of Japanese participants from the SURMOUNT‐1 trial included in the analysis (one study site was excluded due to a regulatory noncompliance issue). ITT, intention‐to‐treat; *N*, number of participants in the analysis population.

Overall, 95.8%, 95.5%, and 93.1% of participants in the tirzepatide 5‐, 10‐, and 15‐mg groups, respectively, completed the study. In the placebo group, 85.2% of participants completed the study. The most common reason for study discontinuation was withdrawal by participant, which occurred for one participant in each of the tirzepatide 5‐mg (4.2%) and 10‐mg (4.5%) treatment groups and for three (11.1%) participants in the placebo group. In the tirzepatide 15‐mg group, two (6.9%) participants discontinued the study—one due to an AE (gastroesophageal reflux disease) and one for an unspecified reason categorized as “other” (Figure 1).

At baseline, participants had a mean (standard deviation [SD]) age of 46.2 (11.5) years, body weight of 87.3 (11.1) kg, waist circumference of 101.4 (8.3) cm, and BMI of 31.8 (3.6) kg/m^2^. Most participants were male (*n* = 66, 64.7%). A total of 67 (65.7%) participants had BMI ≥ 30 kg/m^2^. The mean (SD) duration of obesity was 8.8 (8.5) years, 51 (50.0%) participants had prediabetes (Table 1), and approximately 60% of participants had one or two weight‐related complications (Table S1).

**TABLE 1:** Baseline demographics and clinical characteristics.

Characteristic	Tirzepatide 5 mg (*N* = 24)	Tirzepatide 10 mg (*N* = 22)	Tirzepatide 15 mg (*N* = 29)	Placebo (*N* = 27)	Total (*N* = 102)
Age, years	47.7 (10.8)	45.5 (13.7)	47.8 (10.8)	43.9 (11.0)	46.2 (11.5)
Sex					
Male	15 (62.5%)	15 (68.2%)	19 (65.5%)	17 (63.0%)	66 (64.7%)
Female	9 (37.5%)	7 (31.8%)	10 (34.5%)	10 (37.0%)	36 (35.3%)
Duration of obesity, years	8.2 (7.4)	7.4 (9.0)	9.3 (8.3)	10.0 (9.5)	8.8 (8.5)
Body weight, kg	89.0 (11.1)	86.2 (13.4)	86.6 (9.7)	87.4 (10.8)	87.3 (11.1)
BMI, kg/m^2^	32.5 (4.6)	31.6 (3.4)	31.3 (2.9)	31.9 (3.4)	31.8 (3.6)
BMI category, kg/m^2^					
< 30	7 (29.2%)	8 (36.4%)	10 (34.5%)	10 (37.0%)	35 (34.3%)
≥ 30 to < 35	12 (50.0%)	12 (54.5%)	16 (55.2%)	12 (44.4%)	52 (51.0%)
≥ 35 to < 40	4 (16.7%)	2 (9.1%)	3 (10.3%)	4 (14.8%)	13 (12.7%)
≥ 40	1 (4.2%)	0	0	1 (3.7%)	2 (2.0%)
Waist circumference, cm	103.1 (8.9)	100.2 (8.9)	100.8 (7.4)	101.7 (8.4)	101.4 (8.3)
Blood pressure, mm Hg					
Systolic	126.6 (11.2)	126.2 (12.8)	127.1 (13.6)	123.8 (10.8)	125.9 (12.1)
Diastolic	79.1 (9.1)	79.9 (9.7)	82.2 (10.9)	78.9 (8.9)	80.1 (9.7)
Pulse rate, beats/min	72.0 (9.5)	69.6 (9.7)	73.0 (12.0)	73.2 (8.6)	72.1 (10.0)
Prediabetes	12 (50.0%)	10 (45.5%)	16 (55.2%)	13 (48.1%)	51 (50.0%)
Free fatty acids, mEq/L	0.5 (0.2), *n* = 23	0.5 (0.2), *n* = 21	0.5 (0.1), *n* = 27	0.5 (0.2), *n* = 24	0.5 (0.2), *n* = 95
eGFR, mL/min/1.73 m^2^	95.5 (16.0)	100.6 (14.4)	98.3 (13.7)	101.4 (16.6)	99.0 (15.1)
Lipid levels, mg/dL					
Total cholesterol	202.8 (36.1), *n* = 24	197.6 (27.4), *n* = 21	217.8 (40.0), *n* = 27	204.8 (42.5), *n* = 26	206.3 (37.6), *n* = 98
LDL cholesterol	119.5 (27.8), *n* = 23	121.1 (21.9), *n* = 21	129.4 (34.3), *n* = 27	125.3 (26.6), *n* = 24	124.1 (28.2), *n* = 95
HDL cholesterol	53.0 (10.9), *n* = 23	52.2 (9.8), *n* = 21	57.7 (14.1), *n* = 27	52.1 (13.9), *n* = 24	53.9 (12.5), *n* = 95
Triglycerides	157.8 (70.6), *n* = 24	121.5 (52.5), *n* = 21	153.4 (64.2), *n* = 27	151.4 (77.2), *n* = 26	147.1 (67.7), *n* = 98

*Note*: Data are shown as mean (SD) or *n* (%).

Abbreviations: eGFR, estimated glomerular filtration rate; HDL, high‐density lipoprotein; LDL, low‐density lipoprotein.

### 3.2 Change in Body Weight

**FIGURE 2:**
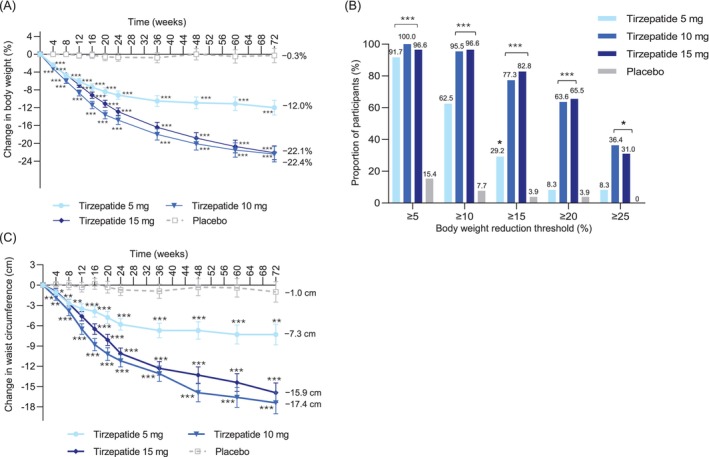
Effect of tirzepatide on body weight and waist circumference. Japanese participants from the SURMOUNT‐1 trial were treated with once‐weekly tirzepatide (5, 10, or 15 mg) or placebo. Data are shown for the modified intention‐to‐treat population (efficacy analysis set). For each parameter, only participants with a nonmissing baseline value and at least one nonmissing postbaseline value were included in the analysis. (A) Percent change in body weight from baseline to week 72 derived from an MMRM analysis. Data are shown as LSM (SE). Change in body weight was statistically significantly lower (*p* < 0.001) from week 4 to week 72 for all tirzepatide treatment groups versus placebo. (B) Proportion of participants achieving body weight reduction thresholds. Results are from logistic regression with missing values imputed by an MMRM analysis. *P* values represent statistically significant differences in the tirzepatide treatment groups versus placebo. (C) Change in waist circumference from baseline to week 72. Data are shown as LSM (SE). Postbaseline data are from an MMRM analysis. LSM, least squares mean; MMRM, mixed model for repeated measures. *p < 0.05 versus placebo, **p < 0.01 versus placebo, ***p < 0.001 versus placebo. [Color figure can be viewed at wileyonlinelibrary.com]

For the first coprimary efficacy endpoint, the least squares mean (LSM) (standard error [SE]) percent change in body weight from baseline to week 72 was −12.0% (1.7%) with tirzepatide 5 mg, −22.4% (1.7%) with tirzepatide 10 mg, −22.1% (1.6%) with tirzepatide 15 mg, and −0.3% (1.6%) with placebo. Compared with placebo, the **percent** change in body weight at week 72 was statistically significantly greater in all tirzepatide treatment groups (*p* < 0.001 for all), with estimated treatment differences (ETD) of −11.7 (95% CI: −16.4 to −7.1) for tirzepatide 5 mg, −22.1 (95% CI: −26.8 to −17.4) for tirzepatide 10 mg, and −21.8 (95% CI: −26.2 to −17.3) for tirzepatide 15 mg (Figure 2A, Table 2).

**TABLE 2:** Primary and secondary endpoints by treatment group.

	Tirzepatide 5 mg (*N* = 24)	Tirzepatide 10 mg (*N* = 22)	Tirzepatide 15 mg (*N* = 29)	Placebo (*N* = 26)	Tirzepatide 5 mg vs. placebo (95% CI); *p*	Tirzepatide 10 mg vs. placebo (95% CI); *p*	Tirzepatide 15 mg vs. placebo (95% CI); *p*
Coprimary endpoints							
Change in body weight (kg)^a^							
Baseline	89.0 (2.3)	86.2 (2.4)	86.6 (2.1)	87.9 (2.2)	ETD: −11.7 (−16.4 to −7.1); *p* < 0.001	ETD: −22.1 (−26.8 to −17.4); *p* < 0.001	ETD: −21.8 (−26.2 to −17.3); *p* < 0.001
Week 72	76.8 (1.5)	68.4 (1.6)	68.5 (1.4)	87.5 (1.5)			
Percent change from baseline to week 72^b^	−12.0 (1.7)	−22.4 (1.7)	−22.1 (1.6)	−0.3 (1.6)			
Participants with ≥ 5% weight reduction^a^, ^b^, ^c^	22 (91.7%)	22 (100%)	28 (96.6%)	4 (15.4%)	OR: 204.3 (16.0–2610.2); *p* < 0.001	OR: 873.6 (22.6–33,817.0); *p* < 0.001	OR: 365.6 (24.1–5558.4); *p* < 0.001
Key secondary endpoints							
Participants with ≥ 10% weight reduction^a^, ^c^	15 (62.5%)	21 (95.5%)	28 (96.6%)	2 (7.7%)	OR: 20.4 (3.9–107.9); *p* < 0.001	OR: 183.0 (18.6–1796.6); *p* < 0.001	OR: 257.6 (27.6–2404.8); *p* < 0.001
Participants with ≥ 15% weight reduction^a^, ^c^	7 (29.2%)	17 (77.3%)	24 (82.8%)	1 (3.9%)	OR: 7.4 (1.1–47.8); *p* = 0.036	OR: 52.9 (7.7–363.6); *p* < 0.001	OR: 81.5 (11.8–563.5); *p* < 0.001
Participants with ≥ 20% weight reduction^a^, ^c^	2 (8.3%)	14 (63.6%)	19 (65.5%)	1 (3.9%)	OR: 1.9 (0.2–15.0); *p* = 0.565	OR: 28.7 (4.4–185.3); *p* < 0.001	OR: 32.8 (5.3–204.9); *p* < 0.001
Waist circumference, cm^a^							
Baseline	103.1 (1.7)	100.2 (1.8)	100.8 (1.6)	101.8 (1.7)	ETD: −6.3 (−10.6 to −2.0); *p* = 0.005	ETD: −16.4 (−20.8 to −12.0); *p* < 0.001	ETD: −14.9 (−19.1 to −10.7); *p* < 0.001
Week 72	94.2 (1.5)	84.1 (1.6)	85.5 (1.5)	100.4 (1.5)			
Change in waist circumference^d^	−7.3 (1.5)	−17.4 (1.6)	−15.9 (1.5)	−1.0 (1.5)			
Additional endpoints							
HbA1c, %^a^							
Baseline	5.5 (0.1)	5.5 (0.1)	5.5 (0.1)	5.7 (0.1)	ETD: −0.5 (−0.6 to −0.4); *p* < 0.001	ETD: −0.7 (−0.8 to −0.5); *p* < 0.001	ETD: −0.7 (−0.8 to −0.6); *p* < 0.001
Week 72	5.1 (0.0)	4.9 (0.0)	4.9 (0.0)	5.6 (0.0)			
Change in HbA1c	−0.5 (0.0)	−0.7 (0.0)	−0.7 (0.0)	0.0 (0.0)			
HbA1c, mmol/mol^a^							
Baseline	37.0 (0.8)	37.1 (0.8)	36.6 (0.7)	38.5 (0.7)	ETD: −5.5 (−6.9 to −4.1); *p* < 0.001	ETD: −7.3 (−8.7 to −5.9); *p* < 0.001	ETD: −7.4 (−8.7 to −6.0); *p* < 0.001
Week 72	31.9 (0.5)	30.1 (0.5)	30.0 (0.5)	37.4 (0.5)			
Change in HbA1c	−5.4 (0.5)	−7.2 (0.5)	−7.3 (0.5)	0.1 (0.5)			
FSG, mg/dL^a^							
Baseline	97.5 (1.8)	95.3 (1.9)	97.8 (1.7)	95.5 (1.7)	ETD: −9.9 (−14.0 to −5.9); *p* < 0.001	ETD: −11.7 (−15.9 to −7.6); *p* < 0.001	ETD: −14.3 (−18.3 to −10.4); *p* < 0.001
Week 72	86.5 (1.4)	84.8 (1.5)	82.1 (1.4)	96.5 (1.4)			
Change in FSG	−10.2 (1.4)	−12.0 (1.5)	−14.6 (1.4)	−0.3 (1.4)			
FSG, mmol/L^a^							
Baseline	5.4 (0.1)	5.3 (0.1)	5.4 (0.1)	5.3 (0.1)	ETD: −0.6 (−0.8 to −0.3); *p* < 0.001	ETD: −0.7 (−0.9 to −0.4); *p* < 0.001	ETD: −0.8 (−1.0 to −0.6); *p* < 0.001
Week 72	4.8 (0.1)	4.7 (0.1)	4.6 (0.1)	5.4 (0.1)			
Change in FSG	−0.6 (0.1)	−0.7 (0.1)	−0.8 (0.1)	−0.01 (0.1)			

*Note*: Data are shown as least squares mean (SE) or *n* (%) for the modified intention‐to‐treat population (efficacy analysis set). All changes are from baseline to week 72. For each parameter, only participants with a nonmissing baseline value and at least one nonmissing postbaseline value were included in the analysis. Analyses were conducted using a mixed model for repeated measures.

Abbreviations: ETD, estimated treatment difference; FSG, fasting serum glucose; HbA1c, glycated hemoglobin; OR, odds ratio.

^a^ *N* = 23 for tirzepatide 5 mg, *N* = 21 for tirzepatide 10 mg, *N* = 24 for tirzepatide 15 mg, and *N* = 22 for placebo at week 72.

^b^ The percent change in body weight in the tirzepatide 5‐mg group was not a coprimary endpoint but was a key secondary endpoint.

^c^ The OR, 95% CI, and *p* value for endpoint measures were from logistic regression analysis with missing data imputed by a mixed model for repeated measures for postbaseline measures.

^d^ The change in waist circumference in the tirzepatide 5‐mg group was not a key secondary endpoint.

For the second coprimary efficacy endpoint, 91.7%, 100%, and 96.6% of participants in the tirzepatide 5‐, 10‐, and 15‐mg groups, respectively, achieved at least a 5% reduction in body weight at week 72 compared with 15.4% of those in the placebo group. Compared with placebo, statistically significantly more participants in the tirzepatide treatment groups (*p* < 0.001 for all) achieved at least a 5% reduction in body weight at week 72, with odds ratios of 204.3 (95% CI: 16.0–2610.2) for tirzepatide 5 mg, 873.6 (95% CI: 22.6–33,817.0) for tirzepatide 10 mg, and 365.6 (95% CI: 24.1–5558.4) for tirzepatide 15 mg (Figure 2B, Table 2).

In addition, compared with placebo, participants in all tirzepatide treatment groups achieved significant body weight reductions of ≥ 10% and ≥ 15%, and participants in the 10‐ and 15‐mg tirzepatide groups achieved a significant weight reduction of ≥ 20% (Figure 2B, Table 2). Treatment with tirzepatide was associated with greater improvements from baseline than with placebo in all key primary and secondary endpoints (Table 2).

### 3.3 Cardiometabolic Risk Factors

Significant reductions in waist circumference from baseline to week 72 were observed in participants treated with any dose of tirzepatide compared with placebo, with LSM (SE) changes of −7.3 (1.5) cm for tirzepatide 5 mg, −17.4 (1.6) cm for tirzepatide 10 mg, −15.9 (1.5) cm for tirzepatide 15 mg, and −1.0 (1.5) cm for placebo (Figure 2C, Table 2).

**FIGURE 3:**
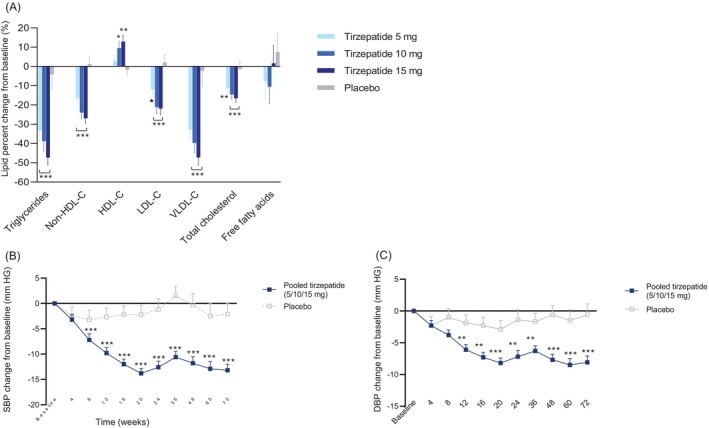
Effect of tirzepatide on lipids and blood pressure. Data are shown for the modified intention‐to‐treat population (efficacy analysis set). For each parameter, only participants with a nonmissing baseline value and at least one nonmissing postbaseline value were included in the analysis. (A) Mean percent change in lipids at week 72 following once‐weekly treatment with tirzepatide (5, 10, or 15 mg) or placebo. Log‐transformed postbaseline data were analyzed with an MMRM. (B) Mean change from baseline to week 72 in SBP using pooled data for the tirzepatide 5‐, 10‐, and 15‐mg groups versus placebo. Data are shown as LSM (SE). Postbaseline data were analyzed with an MMRM. (C) Mean change from baseline to week 72 in DBP using pooled data for the tirzepatide 5‐, 10‐, and 15‐mg groups versus placebo. Data are shown as LSM (SE). Postbaseline data were analyzed with an MMRM. DBP, diastolic blood pressure; HDL‐C, high‐density lipoprotein cholesterol; LDL‐C, low‐density lipoprotein cholesterol; LSM, least squares mean; MMRM, mixed model for repeated measures; SBP, systolic blood pressure; VLDL‐C, very low‐density lipoprotein cholesterol. *p < 0.05 versus placebo, **p < 0.01 versus placebo, ***p ≤ 0.001 versus placebo. [Color figure can be viewed at wileyonlinelibrary.com]

The key secondary and additional endpoints by pooled tirzepatide treatment groups are outlined in Table 3. Compared with placebo, statistically significant differences were observed with tirzepatide treatment from baseline to week 72 for all key secondary endpoints (*p* < 0.01 for all), including **percent change in** triglycerides (ETD: −37.6; 95% CI: −48.0 to −25.2), non‐HDL‐C (ETD: −23.6; 95% CI: −30.5 to −16.0), HDL‐C (ETD: 10.3; 95% CI: 2.7–18.5), and **change in** systolic BP (**mmHG**) (ETD: −11.1; 95% CI: −16.0 to −6.2) (Figure 3A,B, Table 3). In addition, significantly greater decreases from baseline to week 72 were observed with tirzepatide treatment compared with placebo (*p* < 0.001 for all) for the additional endpoints of total cholesterol (ETD: −14.4; 95% CI: −19.4 to −9.0), low‐density lipoprotein cholesterol (ETD: −20.1; 95% CI: −27.4 to −12.0), very low‐density lipoprotein cholesterol (ETD: −39.0; 95% CI: −49.2 to −26.7), and diastolic BP (ETD: −7.5; 95% CI: −11.4 to −3.5) (Figure 3C, Table 3).

**TABLE 3:** Key secondary and additional endpoints by pooled tirzepatide treatment groups.

	Pooled tirzepatide groups	Placebo	ETD from placebo (95% CI); *p*
Key secondary endpoints			
Body weight, kg^a^			
Baseline	86.4 (1.6), *n = 51*	87.9 (2.2), *n = 26*	−10.1 (−12.0 to −8.3); *p* < 0.001
Week 20	76.6 (0.6), *n = 48*	86.7 (0.8), *n = 24*	
Change from baseline to week 20	−10.5 (0.6), *n = 48*	−0.4 (0.8), *n = 24*	
Triglycerides, mg/dL^b^			
Baseline	132.3 (7.3), *n* = 72	133.5 (12.3), *n* = 26	−37.6 (−48.0 to −25.2); *p* < 0.001
Week 72	79.8 (3.7), *n* = 68	128.0 (10.1), *n* = 22	
Percent change from baseline to week 72	−40.3 (2.7), *n = 68*	−4.3 (7.6), *n* = 22	
Non‐HDL cholesterol, mg/dL^b^			
Baseline	149.0 (4.1), *n* = 71	154.0 (7.3), *n* = 24	−23.6 (−30.5 to −16.0); *p* < 0.001
Week 72	115.8 (2.7), *n* = 68	151.6 (6.3), *n* = 22	
Percent change from baseline to week 72	−22.6 (1.8), *n* = 68	1.3 (4.2), *n* = 22	
HDL cholesterol, mg/dL			
Baseline	53.3 (1.4), *n* = 71	50.6 (2.3), *n* = 24	10.3 (2.7–18.5), *p* = 0.008
Week 72	56.9 (1.0), *n* = 68	51.6 (1.6), *n* = 22	
Percent change from baseline to week 72	8.4 (1.9), *n* = 68	−1.7 (3.1), *n* = 22	
Systolic blood pressure, mm Hg^b^			
Baseline	126.7 (1.4), *n* = 75	124.4 (2.4), *n* = 26	−11.1 (−16.0 to −6.2); *p* < 0.001
Week 72	113.1 (1.2), *n* = 68	124.2 (2.1), *n* = 22	
Change from baseline to week 72	−13.2 (1.2), *n* = 68	−2.1 (2.1), *n* = 22	
Additional endpoints			
Total cholesterol, mg/dL^b^			
Baseline	203.9 (4.4), *n* = 72	200.6 (7.2), *n* = 26	−14.4 (−19.4 to −9.0); *p* < 0.001
Week 72	175.1 (2.7), *n* = 68	204.5 (5.4), *n* = 22	
Percent change from baseline to week 72	−14.2 (1.3), *n* = 68	0.2 (2.7), *n* = 22	
LDL cholesterol, mg/dL^b^			
Baseline	120.4 (3.4), *n* = 71	122.3 (5.9), *n* = 24	−20.1 (−27.4 to −12.0); *p* < 0.001
Week 72	98.2 (2.4), *n* = 68	122.8 (5.2), *n* = 22	
Percent change from baseline to week 72	−18.4 (2.0), *n* = 68	2.1 (4.3), *n* = 22	
VLDL cholesterol, mg/dL^b^			
Baseline	61.1 (3.3), *n* = 71	62.1 (5.8), *n* = 24	−39.0 (−49.2 to −26.7); *p* < 0.001
Week 72	36.5 (1.7), *n* = 68	59.9 (4.8), *n* = 22	
Percent change from baseline to week 72	−40.4 (2.7), *n* = 68	−2.4 (7.8), *n* = 22	
Free fatty acids, mEq/L^b^			
Baseline	0.46 (0.02), *n* = 71	0.48 (0.03), *n* = 24	−12.1 (−29.3 to 9.3); *p* = 0.244
Week 72	0.44 (0.02), *n* = 66	0.50 (0.05), *n* = 22	
Percent change from baseline to week 72	−5.5 (5.2), *n* = 66	7.5 (10.2), *n* = 22	
Diastolic blood pressure, mm Hg^b^			
Baseline	80.5 (1.1), *n* = 75	79.4 (1.9), *n* = 26	−7.5 (−11.4 to −3.5); *p* < 0.001
Week 72	72.2 (1.0), *n* = 68	79.7 (1.7), *n* = 22	
Percent change from baseline to week 72	−8.1 (1.0), *n* = 68	−0.6 (1.7), *n* = 22	

*Note*: Data are shown as least squares mean (SE) for the modified intention‐to‐treat population (efficacy analysis set). For each parameter, only participants with a nonmissing baseline value and at least one nonmissing postbaseline value were included in the analysis. Analyses were conducted using a mixed model for repeated measures. Lipids were analyzed with the use of log transformation, and data represent model‐based estimates and 95% CI.

Abbreviations: ETD, estimated treatment difference; HDL, high‐density lipoprotein; LDL, low‐density lipoprotein; VLDL, very low‐density lipoprotein.

^a^ Pooled data for the tirzepatide 10‐ and 15‐mg groups.

^b^ Pooled data for the tirzepatide 5‐, 10‐, and 15‐mg groups.

### 3.4 Safety

At least one treatment‐emergent AE was reported by 75.0%, 63.6%, and 75.9% of participants in the tirzepatide 5‐, 10‐, and 15‐mg groups, respectively, and by 59.3% of participants in the placebo group. Serious AEs were reported by two (8.3%) participants in the tirzepatide 5‐mg group (appendicitis, *n* = 1; prostate cancer, *n* = 1), one (3.4%) participant in the tirzepatide 15‐mg group (uterine leiomyoma), and one (3.7%) participant in the placebo group (dizziness). No deaths were reported. AEs that were considered related to the study treatment as judged by the study investigator occurred in 41.7%, 54.5%, and 51.7% of participants in the tirzepatide 5‐, 10‐, and 15‐mg groups, respectively, and in 11.1% of participants in the placebo group. The most common AEs were constipation, decreased appetite, diarrhea, nausea, injection site reaction, and pyrexia. Aside from constipation, these AEs occurred at a higher frequency with tirzepatide treatment compared with placebo (Table 4).

**TABLE 4:** Summary of adverse events.

	Tirzepatide 5 mg (*N* = 24)	Tirzepatide 10 mg (*N* = 22)	Tirzepatide 15 mg (*N* = 29)	Placebo (*N* = 27)
Participants with ≥ 1 TEAE	18 (75.0%)	14 (63.6%)	22 (75.9%)	16 (59.3%)
Serious AEs	2 (8.3%)	0	1 (3.4%)	1 (3.7%)
Deaths	0	0	0	0
AEs related to study treatment^a^	10 (41.7%)	12 (54.5%)	15 (51.7%)	3 (11.1%)
AEs leading to discontinuation of study	0	0	2 (6.9%)	0
AEs leading to discontinuation of study treatment	1 (4.2%)	0	2 (6.9%)	0
TEAEs occurring in ≥ 2 participants				
Abdominal discomfort	0	1 (4.5%)	4 (13.8%)	0
Abdominal distention	2 (8.3%)	0	0	0
Back pain	2 (8.3%)	1 (4.5%)	1 (3.4%)	1 (3.7%)
Constipation	6 (25.0%)	4 (18.2%)	4 (13.8%)	2 (7.4%)
Decreased appetite	3 (12.5%)	1 (4.5%)	5 (17.2%)	0
Dental caries	1 (4.2%)	2 (9.1%)	1 (3.4%)	2 (7.4%)
Dermatitis contact	0	2 (9.1%)	0	0
Diarrhea	1 (4.2%)	1 (4.5%)	4 (13.8%)	0
Gastroenteritis	1 (4.2%)	2 (9.1%)	0	1 (3.7%)
Gastroesophageal reflux disease	2 (8.3%)	0	1 (3.4%)	1 (3.7%)
Headache	1 (4.2%)	0	3 (10.3%)	0
Injection site reaction	1 (4.2%)	5 (22.7%)	1 (3.4%)	0
Nausea	1 (4.2%)	3 (13.6%)	6 (20.7%)	1 (3.7%)
Pyrexia	2 (8.3%)	3 (13.6%)	4 (13.8%)	3 (11.1%)
Vomiting	0	2 (9.1%)	2 (6.9%)	0
Weight decreased	0	1 (4.5%)	2 (6.9%)	0

*Note*: Data are shown as *n* (%). AEs were coded using the Medical Dictionary for Regulatory Activities (version 24.1) and listed by preferred term.

Abbreviations: AE, adverse event; *N*, number of participants in the analysis population; TEAE, treatment‐emergent adverse event.

^a^ AEs related to study treatment as judged by the investigator.

## 4 Discussion

In conjunction with lifestyle interventions, Japanese adults with obesity or overweight from the SURMOUNT‐1 trial demonstrated significant body weight reductions with all doses of tirzepatide compared to placebo following 72 weeks of treatment. Furthermore, greater body weight reductions were observed with tirzepatide compared with placebo as early as week 4 of treatment. The substantial decrease in body weight observed (12.0%, 22.4%, and 22.1% for tirzepatide 5, 10, and 15 mg, respectively) in this study is comparable to the findings from the global SURMOUNT‐1 trial with body weight reductions of 15.0%, 19.5%, and 20.9% for tirzepatide 5‐, 10‐, and 15‐mg treatment groups, respectively [11]. Similar decreases in body weight were observed in Japanese participants with obesity in the SURMOUNT‐J trial (17.8% and 22.7% for participants receiving once‐weekly tirzepatide 10 and 15 mg, respectively) [10] and in Chinese participants with obesity or overweight in the SURMOUNT‐CN trial (13.6% and 17.5% for participants receiving once‐weekly tirzepatide 10 and 15 mg, respectively) [12].

Overall, the findings in this subpopulation analysis of Japanese participants are consistent with those observed in the total SURMOUNT‐1 population [11]. Baseline characteristics, including age, BP, pulse rate, and renal function, were generally comparable between the Japanese subpopulation and the overall SURMOUNT‐1 population. However, Japanese participants had a lower mean (SD) baseline body weight (87.3 [11.1] vs. 104.8 [22.1] kg) and BMI (31.8 [3.6] vs. 38.0 [6.8] kg/m^2^), and a shorter mean duration of obesity (8.8 [8.5] vs. 14.4 [10.8] years). The proportion of female participants in the Japanese subgroup (35.3% vs. 67.5%) was lower than in the overall population. In contrast, mean (SD) lipid levels at baseline were higher among Japanese participants compared to the overall population: total cholesterol (206.3 [37.6] vs. 187.9 [20.3] mg/dL), LDL cholesterol (124.1 [28.2] vs. 109.5 [30.2] mg/dL), HDL‐C (53.9 [12.5] vs. 47.3 [26.3] mg/dL), and triglycerides (147.1 [67.7] vs. 128.4 [50.0] mg/dL). Additionally, a greater proportion of Japanese participants had prediabetes (50.0% vs. 40.6%), compared with the overall population. Consistent with the findings in the overall SURMOUNT‐1 population, hypertension and dyslipidemia were the most commonly reported baseline comorbidities in the Japanese subpopulation, but with a higher prevalence. Hypertension and dyslipidemia were reported in 41.2% and 67.6% of participants within the Japanese subpopulation, compared to 32.3% and 29.8% in the overall study population, respectively. The prevalence of atherosclerotic cardiovascular disease, obstructive sleep apnea, and osteoarthritis was slightly lower in the Japanese subgroup, reported at 1.0%, 4.9%, and 4.9%, respectively, compared to 3.1%, 7.8%, and 12.8% in the overall population.

The reductions in body weight observed in the current study exceeded the recommended targets for the treatment of obesity. Japanese guidelines recommend dietary, exercise, and behavioral therapies for initial treatment of obesity disease, including a recommended body weight reduction of at least 3% for obesity disease and 5% to 10% for high‐degree obesity disease [2, 13]. In the tirzepatide 5‐, 10‐, and 15‐mg treatment groups, 91.7%, 100%, and 96.6% of participants, respectively, achieved at least a 5% body weight reduction at 72 weeks, with approximately 65% in the 10‐ and 15‐mg groups achieving at least a 20% body weight reduction. Losing 5% to 10% of body weight has been shown to decrease obesity‐related complications and enhance quality of life [14]. Additional weight loss leads to even greater benefits [14, 15].

The body weight reductions observed in Japanese adults were accompanied by significant reductions in cardiometabolic, metabolic, and glycemic parameters, including waist circumference, systolic and diastolic BP, lipids, glycated hemoglobin, and fasting serum glucose. This is consistent with the findings observed in the global population of SURMOUNT‐1 [11] and with the clinically meaningful improvements in several metabolic parameters seen in participants with T2D across the SURPASS clinical program [16, 17, 18, 19, 20], including Japanese participants in the SURPASS J‐mono and J‐combo trials [21, 22, 23, 24]. Improvements in metabolic factors may translate to a reduced risk of developing cardiovascular disease and T2D in Japanese individuals with obesity or overweight. Indeed, in post hoc analyses of SURMOUNT‐1, tirzepatide treatment, compared with placebo, significantly reduced the 10‐year predicted risk of atherosclerotic cardiovascular disease [25] and the 10‐year predicted risk of developing T2D, regardless of baseline glycemic status [26]. In addition, 3‐year efficacy and safety analyses of the global SURMOUNT‐1 trial revealed substantial and sustained weight reduction and a markedly lower risk of progression to T2D with tirzepatide treatment compared with placebo in participants with obesity and prediabetes [27].

The safety profile of tirzepatide in the current study is consistent with findings from the global SURMOUNT‐1 trial and previous findings from the SURMOUNT program in participants with obesity, with or without T2D [10, 11, 12, 28, 29, 30]. Reported treatment‐emergent AEs among Japanese participants within the tirzepatide groups were mostly gastrointestinal‐related, and the incidence of serious AEs was low. No new safety concerns were identified, and most participants treated with tirzepatide completed the study (93%–96%).

Study limitations include the relatively small number of Japanese participants and a limited number of clinical trial sites in Japan from the SURMOUNT‐1 trial. The small number of participants in each treatment arm limits the study's ability to detect subtle dose‐dependent effects; no dose‐dependent decrease in body weight was observed between the tirzepatide 10‐ and 15‐mg doses. However, the consistency of findings with the overall study population, along with the clinically relevant efficacy and safety observed for both doses, supports the use of tirzepatide 15 mg as a viable clinical option when a greater therapeutic effect is desired compared to tirzepatide 10 mg. Of note, dose dependency was observed between the 5‐ and 10/15‐mg doses of tirzepatide, leading to significant and clinically meaningful reductions in body weight within the tirzepatide treatment groups. Additionally, dose‐dependent effects were observed in certain lipid parameters and fasting serum glucose levels. Another limitation is that enrolled participants in the SURMOUNT‐1 trial may have had a greater commitment to weight management compared with the general population. This study did not include participants with T2D; however, data relating to the efficacy and safety of tirzepatide in Japanese people with obesity and T2D from the SURMOUNT‐2 trial are disclosed elsewhere [31]. Despite these limitations, these findings add to the limited data on tirzepatide in Japanese adults with obesity or overweight. Considering the chronic nature of obesity and its related health issues, longer‐term studies of tirzepatide are warranted in this population.

## 5 Conclusion

In conclusion, when used alongside a reduced‐calorie diet and increased physical activity, all three doses of once‐weekly tirzepatide (5, 10, and 15 mg) demonstrated substantial and sustained weight reduction in Japanese adults with obesity or overweight during 72 weeks of treatment compared with placebo. Furthermore, treatment with tirzepatide resulted in improvements in cardiometabolic risk factors. The safety profile of tirzepatide was consistent with previous findings from phase 3 clinical trials.

## Author Contributions

Study conception and design: Tomotoka Shingaki. Data collection: Tomotaka Shingaki and Tomonori Oura. Data analysis: Tomonori Oura. Data interpretation and critical review of the manuscript: all authors. All authors had full access to the data and approved this manuscript to be submitted for publication.

## Funding

This study was sponsored by Eli Lilly and Company.

## Conflicts of Interest

Yasushi Ishigaki has received lecture fees from Eli Lilly Japan K.K., Kowa Co. Ltd., Novo Nordisk Pharma Ltd., and Sumitomo Pharma Co. Ltd. and has received research funding from Daiichi Sankyo, Novo Nordisk Pharma Ltd., and Sanofi. Tomotoka Shingaki and Tomonori Oura are full‐time employees of Eli Lilly Japan K.K. and minor shareholders of Eli Lilly and Company. Iichiro Shimomura has received grants/research funding from Teijin Pharma Ltd., Eli Lilly Japan K.K., Mochida Pharmaceutical Co. Ltd., Kowa Co. Ltd., Kyowa Kirin Co. Ltd., Sumitomo Pharma Co. Ltd., Rohto Pharmaceutical Co. Ltd., Kobayashi Pharmaceutical Co. Ltd., Nissin Food Holdings Co. Ltd., Cancerscan Inc., Mitsubishi Tanabe Pharma Corp., Takeda Pharmaceutical Co. Ltd., Osaka Central Hospital, Hakuhokai Central Hospital, Midori Health Care Center, Kyowakai Medical Corp., Ono Medical Research Foundation, Suzuken Memorial Foundation, Manpei Suzuki Diabetes Foundation, and Japan Agency for Medical Research and Development; consulting fees from MSD K.K., Taisho Pharmaceutical Co. Ltd., Nippon Boehringer Ingelheim Co. Ltd., and Novo Nordisk Pharma Ltd.; and honoraria from ARKRAY Inc., MSD K.K., Astellas Pharma Inc., AstraZeneca K.K., Abbott Japan Co. Ltd., Ono Pharmaceutical Co. Ltd., Otsuka Pharmaceutical Co. Ltd., Kyorin Pharmaceutical Co. Ltd., Johnson & Johnson K.K., Kyowa Kirin Co. Ltd., Kowa Co. Ltd., Sanofi, Sumitomo Pharma Co. Ltd., Sanwa Kagaku Kenkyusho Co. Ltd., Teijin Pharma Ltd., Daiichi Sankyo, Mitsubishi Tanabe Pharma Corp., Chugai Pharmaceutical Co. Ltd., Nipro Corp., Eli Lilly Japan K.K., Nippon Boehringer Ingelheim Co. Ltd., Novartis Pharma K.K., Novo Nordisk Pharma Ltd., Mochida Pharmaceutical Co. Ltd., Bayer Yakuhin Ltd., Pfizer Japan Inc., and Lotte Co. Ltd. Masamichi Yamada declares no conflicts of interest.

## Supporting information


**TABLE S1:** Baseline weight‐related comorbidities.

## Data Availability

Eli Lilly and Company provides access to all individual participant data collected during the trial, after anonymization, with the exception of pharmacokinetic or genetic data. Data are available to request 6 months after the indication studied has been approved in the US and EU and after primary publication acceptance, whichever is later. No expiration date for data requests is currently set once data are made available. Access is provided after a proposal has been approved by an independent review committee identified for this purpose and after receipt of a signed data‐sharing agreement. Data and documents, including the study protocol, statistical analysis plan, clinical study report, and blank or annotated case report forms, will be provided in a secure data‐sharing environment. For details on submitting a request, see the instructions provided at www.vivli.org.
