# Supplementary material for: Efficacy and Safety of Tirzepatide in Japanese Participants With Obesity: A Subpopulation Analysis of the SURMOUNT‐1 Trial
Source: Obesity (Silver Spring). 2026 Jan 30;34(3):608–21. doi: 10.1002/oby.70131 (PMC12933229; doi:10.1002/oby.70131)
Supplement: Supplementary file 1 — TABLE S1: Baseline weight‐related comorbidities. [file OBY-34-608-s001.docx]

**SUPPLEMENTARY MATERIAL**

**Efficacy and safety of tirzepatide in Japanese participants with obesity: Subpopulation analysis of the SURMOUNT-1 trial**

Yasushi Ishigaki^1^ | Masamichi Yamada^2^ | Tomotaka Shingaki^3^ | Tomonori Oura^3^ | Iichiro Shimomura^4^

^1^Division of Diabetes, Metabolism and Endocrinology, Department of Internal Medicine, Iwate Medical University, Iwate, Japan

^2^Tokyo Center Clinic, Tokyo, Japan

^3^Japan Drug Development and Medical Affairs, Eli Lilly Japan K.K., Hyogo, Japan

^4^Department of Metabolic Medicine, Osaka University Graduate School of Medicine, Osaka, Japan

**Correspondence:** Tomotaka Shingaki, Eli Lilly Japan K.K., 5-1-28 Isogami-dori, Chuo-ku Kobe, Hyogo 651-0086, Japan; Tel.: +81-(0)3-5574-9400; Email: [shingaki_tomotaka@lilly.com](mailto:shingaki_tomotaka@lilly.com)

**TABLE S1.** Baseline weight-related comorbidities.

|  | **Tirzepatide 5 mg  (*N* = 24)** | **Tirzepatide 10 mg (*N* = 22)** | **Tirzepatide 15 mg  (*N* = 29)** | **Placebo (*N* = 27)** | **Total  (*N* = 102)** |
| --- | --- | --- | --- | --- | --- |
| **Number of comorbidities** |  |  |  |  |  |
| None | 2 (8.3%) | 3 (13.6%) | 5 (17.2%) | 2 (7.4%) | 12 (11.8%) |
| 1 | 8 (33.3%) | 5 (22.7%) | 7 (24.1%) | 12 (44.4%) | 32 (31.4%) |
| 2 | 6 (25.0%) | 6 (27.3%) | 11 (37.9%) | 4 (14.8%) | 27 (26.5%) |
| 3 | 5 (20.8%) | 5 (22.7%) | 4 (13.8%) | 5 (18.5%) | 19 (18.6%) |
| 4 | 2 (8.3%) | 3 (13.6%) | 2 (6.9%) | 4 (14.8%) | 11 (10.8%) |
| ≥5 | 1 (4.2%) | 0 | 0 | 0 | 1 (1.0%) |
| **Comorbidities^a^** |  |  |  |  |  |
| Hypertension | 11 (45.8%) | 11 (50.0%) | 14 (48.3%) | 6 (22.2%) | 42 (41.2%) |
| Not reported | 13 (54.2%) | 11 (50.0%) | 15 (51.7%) | 21 (77.8%) | 60 (58.8%) |
| Dyslipidemia | 17 (70.8%) | 12 (54.5%) | 19 (65.5%) | 21 (77.8%) | 69 (67.6%) |
| Not reported | 7 (29.2%) | 10 (45.5%) | 10 (34.5%) | 6 (22.2%) | 33 (32.4%) |
| Atherosclerotic CVD | 0 | 0 | 1 (3.4%) | 0 | 1 (1.0%) |
| Not reported | 24 (100%) | 22 (100%) | 28 (96.6%) | 27 (100%) | 101 (99.0%) |
| Obstructive sleep apnea | 1 (4.2%) | 2 (9.1%) | 0 | 2 (7.4%) | 5 (4.9%) |
| Not reported | 23 (95.8%) | 20 (90.9%) | 29 (100%) | 25 (92.6%) | 97 (95.1%) |
| Osteoarthritis | 1 (4.2%) | 2 (9.1%) | 0 | 2 (7.4%) | 5 (4.9%) |
| Not reported | 23 (95.8%) | 20 (90.9%) | 29 (100%) | 25 (92.6%) | 97 (95.1%) |
| Anxiety/depression | 0 | 0 | 0 | 1 (3.7%) | 1 (1.0%) |
| Not reported | 24 (100%) | 22 (100%) | 29 (100%) | 26 (96.3%) | 101 (99.0%) |
| MASLD | 5 (20.8%) | 6 (27.3%) | 5 (17.2%) | 7 (25.9%) | 23 (22.5%) |
| Not reported | 19 (79.2%) | 16 (72.7%) | 24 (82.8%) | 20 (74.1%) | 79 (77.5%) |
| Asthma or COPD | 2 (8.3%) | 2 (9.1%) | 3 (10.3%) | 3 (11.1%) | 10 (9.8%) |
| Not reported | 22 (91.7%) | 20 (90.9%) | 26 (89.7%) | 24 (88.9%) | 92 (90.2%) |
| Gout | 11 (45.8%) | 9 (40.9%) | 7 (24.1%) | 9 (33.3%) | 36 (35.3%) |
| Not reported | 13 (54.2%) | 13 (59.1%) | 22 (75.9%) | 18 (66.7%) | 66 (64.7%) |

Note: Data are shown as *n* (%).

Abbreviations: COPD, chronic obstructive pulmonary disease; CVD, cardiovascular disease;, MASLD, metabolic dysfunction-associated steatotic liver disease

^a^Baseline comorbidities were based on medical history terms and collected via case report forms.
